# IgG型淋巴浆细胞性淋巴瘤合并继发性Fanconi综合征一例

**DOI:** 10.3760/cma.j.issn.0253-2727.2021.07.015

**Published:** 2021-07

**Authors:** 椋 王, 晓月 王, 慧芳 丁

**Affiliations:** 1 胜利油田中心医院血液内科，山东省东营市 257034 Department of Hematology, Shengli Oilfield Central Hospital, Dongying 257034, China; 2 潍坊医学院临床医学院，潍坊 261000 School of Clinical Medical College, Weifang Medical University, Weifang 261000, China

患者，男，66岁，2019年12月无诱因出现双膝关节疼痛，活动后加重，休息后略缓解。2020年4月入住胜利油田中心医院风湿免疫科，查HLA-B2704阳性，行PET-CT检查提示为骶髂关节融合，诊断为“强直性脊柱炎”，予“锝-亚甲基二磷酸盐”等治疗后症状缓解。后症状逐渐加重，累及双踝关节、双肩关节，2020年6月再次于我院治疗。生化常规：碱性磷酸酶175 U/L，钙2.08 mmol/L，磷0.58 mmol/L，LDH 102.00 U/L，葡萄糖4.76 mmol/L，尿酸107.40 µmol/L，钾2.95 mmol/L，碳酸氢盐8.30 mmol/L，β_2_-微球蛋白4.41 mg/L。尿常规：尿葡萄糖（++），尿蛋白（++）。24 h尿蛋白定量5.85 g/24 h，尿蛋白1.89 g/L。风湿检查：IgM 0.39 g/L，IgG 18.58 g/L，IgA0.52 g/L，C_3_ 0.51 g/L，C_4_ 0.06 g/L。轻链κ、λ定量：κ 6.36 g/L，λ 1.00 g/L，κ/λ 6.38。肩关节MRI：右侧肩袖部分损伤；肩关节腔、喙突下滑囊、肩峰下滑囊少量积液。以浆细胞病转入我科。查体：左侧肩关节压痛，外展及屈伸受限，双膝关节可触及骨擦感，浮髌征阴性，双下肢凹陷性水肿。动脉血气分析：pH 7.24，标准碳酸氢盐18 mmol/L，氯离子浓度115 mmol/L，乳酸浓度4 mmol/L。24 h尿钙：0.61 g；24 h尿钾：4.80 g。血清游离轻链：κ轻链3900 mg/L，λ轻链20.50 mg/L，κ/λ 190.24。尿游离轻链：κ轻链6450 mg/L，λ轻链70.70 mg/L，κ/λ 91.23。血清、尿免疫固定电泳：IgG泳道发现沉淀条带，κ泳道发现两条沉淀条带，免疫球蛋白类型为IgG-κ伴κ游离轻链型。血清M蛋白定量：7.5 g/L。腹部超声：脾大，厚度45 mm，长径126 mm。PET-CT：双侧膝关节积液；双侧小腿及足部皮下水肿；右肩关节、双髋关节及膝关节周围软组织炎性改变。骨髓细胞学检查示：增生活跃，淋巴细胞比例增高（30％），部分细胞胞质边缘有绒毛状突起，意见：三系增生，淋巴细胞增多原因待查。骨髓免疫学分型：可见9.35％异常B淋巴细胞，其免疫表型CD19（+），CD20（+），CD38（−），胞内免疫球蛋白κ轻链限制性表达，提示为单克隆B细胞；另可见浆细胞占有核细胞总数0.10％，其免疫表型为CD27（+），CD28（+），CD38（+），CD19（部分+），CD138（部分+），CD20（−），CD56（−），CD117（−），胞内免疫球蛋白κ轻链限制性表达，提示为单克隆浆细胞。骨髓活检：骨髓有核细胞增生程度较活跃（造血面积约70％）；粒/红比例大致正常；粒系以偏成熟阶段细胞为主，偏幼稚细胞散在少数；红系以中晚幼红细胞为主；巨核细胞数量在正常范围，以分叶核为主；淋巴细胞散在分布，浆细胞易见；骨髓间质未见胶原纤维增生。免疫组化：CD38（小簇及散在少量+），CD138（少量+），CD56（偶见+），CD20（灶性及散在+），PAX-5（小簇及散在少量+），Ki-67（<10％+），MUM-1淋巴细胞（−），CD10（−），考虑B细胞肿瘤，建议行MYD88基因检查除外淋巴浆细胞淋巴瘤（LPL）可能。成熟B细胞淋巴瘤相关基因突变检测：CXCR4 S338突变阳性，突变频率3.20％；MYD88 L256P突变阳性，突变频率4.80％；KMT2C突变阳性，突变频率6.20％。染色体核型分析：核型46，XY[20]。诊断为：IgG型LPL、继发性范可尼综合征、强直性脊柱炎。

患者2020年7月应用BDR方案（硼替佐米+地塞米松+利妥昔单抗）治疗3个疗程，并予对症支持治疗，骨痛症状消失。2020年10月复查骨髓形态学、免疫学分型未见异常。血清、尿免疫固定电泳均为阳性（IgG-κ伴κ游离轻链型）。MYD88基因L265P突变检测阳性（突变频率0.10％）。血清游离轻链：κ轻链2362.50 mg/L，λ轻链16.00 mg/L，κ/λ 147.66。血清M蛋白定量5.50 g/L。根据2020版NCCN指南，该患者疗效评价为微小缓解。第3个疗程化疗结束后，患者出现带状疱疹病毒感染，并遗留神经痛。2020年10月患者应用ZR方案（泽布替尼+利妥昔单抗）治疗3个疗程，复查血清免疫固定电泳：SP（+），IgG（+），κ轻链（+）。尿免疫固定电泳：SP（+），IgG（−），κ轻链（+）。尿免疫球蛋白轻链定量：κ轻链210.60 mg/L，λ轻链<25.00 mg/L。血清游离轻链：κ轻链762.60 mg/L，λ轻链12.40 mg/L，κ/λ 61.50。血清M蛋白定量：2.50 g/L，疗效评价为部分缓解。随访至2021年2月，患者一般情况可，双下肢轻度水肿，无其他明显不适，拟继续应用ZR方案维持治疗。

